# Comparative study of the registration procedure of locally manufactured herbal products in selected African countries

**DOI:** 10.3389/fphar.2025.1695809

**Published:** 2025-12-05

**Authors:** Aisha Gambo, Nceba Gqaleni, Limakatso Lebina, Lehlohonolo J. Mathibe

**Affiliations:** 1 Sub-Discipline of Traditional Medicine, School of Medicine, College of Health Sciences, University of KwaZulu-Natal, Durban, South Africa; 2 Africa Health Research Institute, KwaZulu-Natal, South Africa; 3 Discipline of Pharmaceutical Sciences, School of Health Sciences, University of KwaZulu-Natal, Durban, South Africa

**Keywords:** registration, locally manufactured herbal medicines, African countries, indigenous medicinal plants, registration requirements

## Abstract

**Background:**

Recognizing the widespread use of herbal medicines, regulating them to ensure their safe registration, quality, and efficacy is of vital importance. Various countries in Africa have developed guidelines for the registration and subsequent market authorization of herbal medicine products. In this article, we aim to examine the registration procedures of locally manufactured herbal medicine (LMHM) products in four selected African countries for comparative purposes.

**Method:**

Document analysis was used to evaluate and interpret official and legal documents identified from the official websites of the drug regulatory authorities (DRAs) in the selected countries [Ghana, Nigeria, Uganda, and Zimbabwe]. These countries are selected based on the study’s objectives to examine the regulatory framework, guidelines, and legislation on the registration procedures and requirements of LMHMs.

**Results:**

In Ghana, Nigeria, and Uganda, the registration process offers a simplified pathway for LMHMs before market authorization. In Nigeria, LHMHs are listed, whereas in both Ghana and Uganda, they are notified. In Zimbabwe, herbal products are registered as either complementary medicines for general sale or pharmacy complementary medicines. In Nigeria and Uganda, the herbal product will undergo 2 years of pharmacovigilance for post-market surveillance after approval. Products are recalled when complaints occur during pharmacovigilance. In Ghana, yearly and periodic renewals are being done for already registered products. In Uganda, after 2 years of product tracking, the herbal product is then fully registered with the National Drug Authority. The minimum requirements for registering herbal medicines are to ensure product safety, efficacy, and quality. In all selected countries, emphasis is placed on safety and quality. Additionally, experiences reported by physicians, traditional practitioners, or treated patients also serve as evidence for therapeutic efficacy. In all countries, herbal products must conform to existing labeling regulations.

**Conclusion:**

The registration of LMHMs is simplified in selected African countries. However, despite this flexibility, few LMHM products get registered and obtain market authorization. Drug regulatory authorities in Africa must address the challenges that hinder the registration of herbal products and take action to promote the local production of herbal medicines while complying with the fundamental registration criteria for product safety, efficiency, and quality control.

## Background

Herbal medicine, which comprises crude plant materials, herbal preparations, and finished products that contain plant parts as active ingredients, is the most popular and lucrative form of traditional medicine. It is the oldest and most widely practiced system of medicine in the world (Asase, 2023). Despite advancements in biomedical practice, approximately 88% of countries globally were estimated to use herbal medicine to cater to their health-related needs (WHO, 2022). This could be due to the positive attributes of herbal medicine, which include its diversity, flexibility, ease of accessibility, increased acceptability, affordability, and increased economic importance (Payyappallimana, 2010).

Literature has shown that, regardless of the positive attributes and the vital role of herbal medicine, there have been major safety concerns associated with the use of herbal medicines that are unregistered, whose safety and efficacy have not been evaluated (Usai et al., 2021). There is a general belief that herbal medicines are safe, harmless, and free from any side effects (van Wyka and Prinsloo, 2020). However, numerous scientific studies have reported that genotoxic, mutagenic, or carcinogenic effects are associated with the use of herbal medicine (Okaiyeto and Oguntibeju, 2021; Van Wyka and Prinsloo, 2020). Renal and kidney dysfunction, stomach upsets, and diarrhea were reported due to uncontrolled consumption of herbal medicines (Okaiyeto and Oguntibeju, 2021).

Additionally, poisoning due to the use of herbal medicines is not rare (Van Wyka and Prinsloo, 2020). Several studies have reported the presence of high concentrations of heavy metals, including lead, mercury, and arsenic, in certain herbal medicines. A study reported that out of 247 traditional Chinese medicines assessed, 5%–15% contained arsenic, 5% contained lead, and 65% contained mercury (Sakharkar, 2017). The lack of appropriate quality control measures, inadequate labeling, and the absence of suitable patient information further compromise the safety of herbal medicines (Ekor, 2014). Therefore, the regulation of herbal medicines to ensure that they are appropriately registered for their assured safety, quality, and effectiveness for public health cannot be overemphasized.

Traditional medicines are broadly categorized into four main groups. Category 1: traditional medicines that have been extemporaneously prepared by traditional health practitioners for treating patients using raw materials that are fresh or dry, which are generally short-lived. The safety and efficacy of the medicines are justified by a long period of use. This category of traditional medicines is not directly controlled by national drug regulatory agencies. Traditional medicines in category 2 are those obtained from the traditional pharmacopeia, widely used in the community. They are prepared in advance with a well-known formulation, whose active ingredients are raw materials. The safety and efficacy of the medicines are justified by the ethnomedical evidence of a long history of use or by open clinical trials (CTs), if deemed necessary by the authority. However, if medicines from category 2 were to be made available on the market, they would need to meet the general minimum requirements for the registration of traditional medicines. Category 3 traditional medicines are those developed through scientific research. The formulation is prepared in advance, containing active ingredients that are standardized extracts. The safety and efficacy are guaranteed by preclinical and clinical trials conducted using approved standard protocols. However, if medicines from category 3 were to be made available on the market, they would need to meet the general minimum requirements for the registration of traditional medicines. Finally, the traditional medicines in category 4 are those developed through scientific research, containing purified molecules as active ingredients. The safety and efficacy are guaranteed by preclinical and clinical trials conducted using approved standard protocols (Sangho et al., 2024b; WHO, 2010).

The regulatory status of herbal medicines differs from one country to another. In Africa, each region has its unique herbal medicines that form part of a holistic traditional medicine. They involve the use of local plants that are indigenous to that region. The knowledge and practices are passed on verbally from one generation to the next as the knowledge is developed over many centuries through trial and error (Mikhail et al., 2017). These indigenous herbal medicines are, therefore, different from complementary or alternative medicines (CAMs) as these refer to a broad set of healthcare practices that are not part of that country’s tradition or conventional medicine and are not fully integrated into the established healthcare system (WHO, 2024). It forms an aspect of medicine that has been adopted by other populations outside its indigenous culture (Mikhail et al., 2017). These medicines include aromatherapy, ayurveda, homeopathy, traditional Chinese medicines, and Western herbal medicines (Sahpra, 2024).

In this article, we focus on the registration of those herbal products that are locally manufactured using indigenous medicinal plants in various regions in Africa. The emphasis is on categories 2 and 3 traditional medicines as these categories get submitted to the regulatory authorities for registration and subsequent market approval. These products are termed locally manufactured herbal medicines (LMHMs).

Various countries in Africa have different regulations on how to register LMHM and CAM, with each country adopting a registration process deemed most compatible with local requirements. Ideally, there should be separate regulations on the registration procedures and requirements for the LMHM and CAM based on the key differences between them (Table 1).

**TABLE 1:** Key differences between locally manufactured herbal medicines and complementary and alternative medicines.

Locally manufactured herbal medicines (LMHMs)	Complementary and alternative medicines (CAMs)
Rooted in traditional practices and may have cultural significance	Often align with mainstream healthcare systems and may have more scientific evidence
Lack of standardization due to variability in quality	Standardized and often regulated
Highly accessible and readily available	Not readily available
Indigenous herbal products with lesser regulatory standards	Mostly imported from countries with higher regulatory standards
Lesser superiority in terms of production, presentation, and global market coverage	Higher superiority in terms of production, presentation, and global market coverage

LMHM, which is mostly based on traditional knowledge and practices that are passed down through generations within a community, is regarded as having a long history of use, which serves as evidence of its safety and efficacy. A long history of use is often defined as a period of use of an herbal product that ranges from 20 to 30 years. Although there is variation in the range of the periods between countries and cultures, the evaluation of the safety and effectiveness of herbal products is mainly dependent on experience (Usai et al., 2021).

In the African region, few studies reported on the approximate number of years for an herbal medicine to qualify for a long history of use. In Uganda, it was reported in the guidelines for the regulation of local herbal medicines that for traditional medicines under category 1 and category 2 classification, their safety and efficacy should be justified by a long period of use, at least more than 10 years (NDA, 2021). However, literature has shown that traditional herbal medicine usage has a very long historical background that corresponds to the Stone Age (Ozioma and Chinwe, 2019).

In some European countries, the safety and evaluation of herbal product effectiveness have been ascertained based on proof of a long history of use and traditional experiences, whereas proof from clinical studies may be necessary in other countries (Usai et al., 2021). An herbal product must be recognized in the literature or demonstrated by ethnopharmacological or ethnobotanical studies to be safe to be categorized as having a long history of use. Thereafter, approval for product commercialization can be obtained without the provision of safety and efficacy data from preclinical or clinical studies (Moreira et al., 2014) (Table 2).

**TABLE 2:** Documentation of safety based on experience (WHO, 2010; Usai et al., 2021).

Condition	Action required
No toxicological data	Assess risk based on documented evidence of traditional use and experience (for at least 20 years) without any reported adverse events. The period of drug use must be considered
Some toxicological data exist	Document condition/s treated, figure of treated patients, and place of treatment
There is toxicity	Try to show the relation to the dose administered
There is potential misuse	Document all misuse and dependence cases
There is no proof of long-term traditional use	Toxicological studies should be done

However, there are situations in which LMHMs should be rigorously regulated. This includes LMHM for which the safety as well as the efficacy profile is not known; when LMHM is claimed to be used for indications different from those for which it is known; when it is used in doses or in populations different from those for which it is commonly used; and when LMHM is used in combination with other herbal drugs whose uses and safety are not established (NAFDAC, 2024). These situations demand a more stringent regulatory process to ascertain the claims, safety, and efficacy of the product. This is usually achieved by conducting clinical trials on the herbal product (WHO, 2005). As part of the regulatory framework, a clinical trial serves as a registration requirement for LMHM having abovementioned properties.

CTs are defined as the studies of new tests and treatments and the evaluation of their effects on human health outcomes using approved guidelines (WHO, 2022). In 2019, Kasilo reported significant progress in the research and development of ATM that was undertaken by countries in the WHO African region. The report highlighted the crucial role of clinical trials in the development of standardized phytomedicines that are safe, efficacious, and of optimum quality (Kasilo et al., 2019). Clinical trials for herbal medicines can be challenging due to their complex composition and potential for individualized formulations as herbal preparations are often made based on individual patient needs. Difficulties in creating effective placebos and a lack of robust scientific evidence further complicate the process. Resource constraints for conducting clinical trials, which include limited funding, infrastructure, and personnel, are a major challenge, particularly in developing countries (Parveen et al., 2015; Walters, 2014; Sangho et al., 2024a; Koonrungsesomboon et al., 2024). As a result of these challenges, few clinical trials are conducted for herbal medicines in Africa. An effective regulatory framework for clinical trials for herbal medicines should be developed, taking into consideration their uniqueness and challenges.

Therefore, based on the aforementioned factors, it is pertinent to report that several key factors come into play in ensuring herbal medicine products are appropriately registered for their assured safety, quality, and efficiency before they are commercialized for public consumption.

In this paper, we aim to examine the regulations governing the registration of locally manufactured herbal medicinal products in selected African countries and make a comparison.

## Study methodology

### Study design

Document analysis was used for data selection of official and legal documents from official websites of the drug regulatory authorities (DRAs) and from other relevant data sources in the selected countries [Ghana, Nigeria, Uganda, and Zimbabwe] for the regulatory framework, guidelines, legislations, and various data sources on the registration procedures and requirements of locally manufactured herbal medicines.

### Study targets

Geopolitically, Africa is often divided into five main regions: North Africa, West Africa, Central Africa, East Africa, and Southern Africa (WorldAtlas, 2025). The study targeted two countries from Western Africa (Ghana and Nigeria) and two countries from Central and Southern Africa, respectively (Uganda and Zimbabwe). These four countries were selected based on the study’s objectives.

### Data collection and analysis

Document analysis was used for data selection/collection. Document analysis is a form of the qualitative research method that involves a systematic review and evaluation of documents, followed by interpretation by the research worker to gain understanding and provide meaning to a research topic (Bowen, 2009). The analytical framework implemented in the study includes the preparation of materials and the extraction of data. This involves formulating categories for data collection and interpretation. An analysis of the content was conducted on the documents, followed by a synthesis of the study findings (Williams, 2025).

Official and legal documents from official websites of the drug regulatory authorities in the selected countries [Ghana, Nigeria, Uganda, and Zimbabwe] were identified for the regulatory framework, guidelines, and legislation on the registration procedures and requirements of LMHMs. Other relevant documents that address the research topic from other sources were also identified. Additionally, focal persons from drug regulatory authorities in the selected countries were briefly consulted to validate the authenticity of the data that were obtained. Inclusion and exclusion criteria used for the data search are presented in Table 3.

**TABLE 3:** Inclusion and exclusion criteria for data used in the document analysis.

Inclusion criteria	Exclusion criteria
Documents available in English	Documents available in other languages
In herbal products, the active ingredient(s) are purely and naturally derived plant substance(s) and are responsible for the overall therapeutic effect of the product	Other types of products/preparation, including cosmetics, medical devices, or conventional medicines
Herbal products manufactured locally (in the country of origin)	Herbal products imported from other countries, including complementary and alternative medicines (CAMs)
Herbal products that are used for the curing and treatment of ailments	Herbal products that are used for other purposes, such as culinary or cosmetic purposes
Herbal preparations for human use only	Herbal preparations for veterinary purposes

Based on the aim of the study, the criteria for comparison between the four selected African countries include registration procedures [application procedure, supporting documents needed, evaluation process, registration category, and post-registration procedure] and registration requirements [minimum requirements for safety, efficacy, product quality, and product label]. Therefore, the first category used for the selection of data was the “registration procedure for LMHMs” in each of the selected countries. The second category was the “main registration requirements of herbal medicines.” The third category concerned the “regulation for the conduct of clinical trials of herbal medicine.”

The data input was done in a Microsoft Excel spreadsheet version 2505. Data were presented in a comparison table and were analyzed for similarities and differences across the selected countries.

## Results

Regulatory approaches for the registration of locally manufactured herbal medicine products in the selected countries were assessed using their websites and published literature. Table 4 summarizes the comparison of the registration procedures of the four African countries that were analyzed.

**TABLE 4:** Summary of a comparison of the registration procedures of locally manufactured herbal medicines (LMHMs) in selected African countries.

Step/procedure	Countries and description of the registration procedure			
	Ghana	Nigeria	Uganda	Zimbabwe
Step 1	1. Pre-analysis • Before registration, the product undergoes pre-analysis at accredited institutions such as the Centre for Plant Medicine Research in Mampong (CPMR), the University of Ghana, and the Kwame Nkrumah University of Science and Technology (KNUST) • These analyses include toxicology, microbiology, and phytochemical testing	1. Business registration and facility inspection • Business registration A valid business registration (e.g., Certificate of Incorporation or Business Name) is required for all applicants, including MSMEs • GMP compliance The herbal medicine production facility must meet good manufacturing practice (GMP) standards, including adequate laboratory facilities for quality control • Production inspection As part of the registration process of herbal medicine and related products, relevant information on production facilities is provided to NAFDAC, and the application for inspection of a facility for compliance with good manufacturing practices (NAFDAC., 2021b) is submitted along with supporting documents including evidence of business registration, trademark acceptance, and artwork of labels	1. Product validation • Safety assessment: the herbalist should have the product validated, usually by the National Chemotherapeutics Research Institute (NCRI) • Testing: this includes phytochemical screening to identify medicinal components, toxicity tests to ensure that the product is safe for human consumption, and other relevant laboratory analyses • Documentation: the herbalist should maintain detailed records of the plant sources, preparation methods, and test results	1. Application requirements • The procedure for obtaining approval in Zimbabwe involves the completion of the statutory application CM-1 Form together with a dossier of all supporting documents
Step 2	2. Application submission • After obtaining the necessary analysis results, the applicant can download the application form from the Food and Drugs Authority (FDA) website or collect it directly from the FDA premises • The form requires detailed information about the product, and the manufacturer gathers all necessary documents, including product details, ingredients, manufacturing process, and safety data	2. Application process • Online application Obtain the required technical information templates [National Agency for Food and Drug Administration and Control Herbal Medicines Technical Information submission template (HMTIST)] from the link provided on the NAFDAC website to guide the preparation of the technical information needed for submission • Written application Submit a written application on the company’s letterhead, addressed to the Director-General of NAFDAC. • Supporting documents Include two sets of the following documents with the application • Evidence of business incorporation or name • Evidence of payment to NAFDAC • Contract manufacturing agreement (if applicable) • Completed Herbal Medicine Technical Information Submission Template (HMTIST) • Submission Submit the application letter, printed registration form, and supporting documents. In addition, herbal product samples will be submitted for assessment	2. Notification with the NDA • Application: a 1-page simplified notification format is filled out by the herbalists to the National Drug Authority (NDA) for notification • Required information: provide all necessary details about the product, including its composition, manufacturing process, and intended use	2. Payment procedure and application submission • Completion of an EVR Quotation Confirmation Form. The form contains, among other information, fees and banking information, which is divided into locally manufactured or externally manufactured products. • Upon successful payment, a specific job invoice is then generated using the EVR Quotation Confirmation Form, and an application can be submitted • Submission procedure The following documents and items are required for the submission procedure: 1. An MCAZ job invoice issued by the Finance Unit. 2. A fully completed and signed CM-1 Form 3. A dossier of supporting documents in the appropriate format and presentation. 4. Appropriate samples for each application as indicated in the guidelines and submitted through the sample repository office. 5. Complete the sample submission form
Step 3	3. FDA evaluation • Once submitted, the application undergoes a thorough evaluation by the FDA, which includes a confirmatory test to validate the product’s safety and quality • A notification is communicated to the applicant within 15 working days to acknowledge receipt of the application • If there are any queries or issues identified, the applicant may be asked to provide additional information or adjust the product during the process of evaluation • The outcome of the evaluation may result in the product being approved or rejected • The FDA will evaluate the product for safety, quality, and efficacy	3. NAFDAC review and approval • Document review: the NAFDAC will review the submitted documents and the technical information provided • GMP inspection: a GMP inspection of the production facility is conducted • Laboratory analysis: laboratory analysis of the product will be conducted at the NAFDAC-designated laboratories in Lagos or Kaduna States	3. NDA review and verification (follow-up): the NDA will review the application and verify the information submitted by the herbalists with the NCRI. The NDA may request further information or documentation • All premises manufacturing and engaged in the sale of herbal medicines must be inspected by NDA and issued with a certificate of suitability of premises (NDA., 2017) • The herbal product is then notified by the NDA and allowed to be commercialized	3. Screening and evaluation • All applications undergo screening within 10 working days of submission. Applications that have passed the screening will proceed to the evaluation stage • Inspectors evaluate company conformance to current good manufacturing practices (GMPs) and may conduct GMP inspections before product registration • A maximum of two review cycles are considered during the evaluation process of applications
Approval and registration/listing/notification of product	4. Approval and surveillance • If the product is approved, it is subject to periodic surveillance by the FDA. • This monitoring ensures continued compliance with safety standards • Once approved, the applicant will receive a license to market the herbal product in Ghana	4. FDRC approval: products that meet the requirements are presented to the Food and Drug Registration Committee (FDRC) for approval (NAFDAC, 2021c) • Registration (listed): upon approval, the herbal product is listed; it is provided an NAFDAC registration number with a capital letter L at the end, indicating a listed herbal product. A Certificate of Product Registration is issued • Herbal products must have a disclaimer “These claims have not been evaluated by NAFDAC’’ on the product label alongside the NAFDAC registration number • The herbal product is released for commercialization	4. NDA approval: the herbal product is then notified by the NDA and allowed to be commercialized	4. Review cycles/final regulatory decision • A maximum of two review cycles will be considered during the evaluation process of applications before the authority reaches a final regulatory decision, which involves the issuance of a registration certificate
Pharmacovigilance/product surveillance	5. Approval and surveillance • The approved product is subjected to periodic surveillance by the FDA • This monitoring ensures continued compliance with safety standards • Once approved, the applicant will receive a license to market the herbal product in Ghana 6. New registration and renewals • A new product registration could take approximately 3 months • Yearly and periodic renewals are also being done for already registered products, which also take approximately a month. In both cases, the time specified holds if there are no issues with the product	5. Pharmacovigilance: thereafter, the herbal product will undergo 2 years of pharmacovigilance for post-market surveillance, where products are monitored for adverse effects, safety, efficacy, and quality. Products are recalled when complaints occur during pharmacovigilance • Every herbal product registered (listed) in Nigeria must renew its registration after 2 years • As a law, all listed herbal products with the capital letter L alongside their NAFDAC registration number cannot be exported outside Nigeria	5. Post-notification • Continuous monitoring: after notification, additional information on product safety and efficiency is required to be submitted by the herbalist by tracking the product for 2 years before product registration by the NDA. • Compliance: follow all regulations and guidelines set by the NDA for the manufacturing and sale of herbal medicines • After 2 years, if the product receives positive tracking results on safety and efficiency, the herbal product will be fully registered with the NDA. The herbalist fills out relevant forms with their details and information on the herbal product to meet the minimum national registration requirements (CCFU., 2008)	

In the following sections, we will analyze the drug regulatory authority of each country concerning the registration of locally manufactured herbal medicines.

### Ghana

In Ghana, the Food and Drugs Authority (FDA) (https://fdaghana.gov.gh/) is the regulatory body responsible for the provision and enforcement of standards for the manufacture, import, export, sale, and distribution of food, drugs (herbal/allopathic/veterinary), cosmetics, and other regulated products, and the regulation of clinical trials to safeguard public health and safety (FDA, 2025). Established in 1997, the FDA draws its mandate from Parts 6, 7, and 8 of the Public Health Act, 2012 (Act 851) (FDA, 2025). The Act prohibits the sale of unregistered herbal products and mandates their proper product registration to ensure public safety and protect health (MOH, 2012).

The Drug and Herbal Medicine (DHM) Registration Directorate of the FDA’s key regulatory functions includes the registration and regulation of allopathic drugs, food supplements, and herbal and homoeopathic products (FDA, 2025).

The Traditional and Alternative Medicine Directorate (TAMD) of the Ministry of Health (MoH) is involved in the development of policies, plans, regulations, and standards of traditional and alternative medicine practices (TAMD, 2025). Together with the FDA, the two organizations work to regulate and promote the use of traditional and alternative medicines in Ghana. The FDA focuses on the safety and quality of products, and the TAMD focuses on the practices and integration of traditional medicine into the healthcare system (MOH, 2005; FDA, 2025).

### Nigeria

The regulation of herbal medicine in Nigeria is governed by several regulatory bodies, including the National Agency for Food and Drug Administration and Control (NAFDAC) (https://nafdac.gov.ng/), alongside the Traditional, Complementary, and Alternative Medicines Department under the Federal Ministry of Health and Social Welfare (FMOH, 2025; NAFDAC, 2025). The NAFDAC aims to supervise the manufacture, importation, distribution, sale, and use of herbal products in Nigeria through the development of established guidelines and regulatory documents to ensure that they meet safety, efficacy, and quality standards (NAFDAC, 2020b). The agency draws its mandate under the Herbal Medicines and Related Products (Registration) Regulations, 2021, by sections 5 and 30 of the National Agency for Food and Drug Administration and Control Act (Cap NI LFN) 2004 and section 12 of the Food, Drug, and Related Products (Registration, etc.) Act Cap F33 LFN 2004 (NAFDAC, 2021d). In Nigeria, the registration of all herbal medicines, including LMHM and CAM, is conducted by the NAFDAC, and they both have the same registration regimen.

The Traditional, Complementary, and Alternative Medicines Department under the Federal Ministry of Health and Social Welfare is responsible for promoting the safe and effective use of traditional medicine, alongside conventional healthcare, focusing on policy development, regulation, and provision of education on traditional, complementary, and alternative medicine (FMOH, 2025).

### Uganda

The National Drug Authority (NDA) (https://www.nda.or.ug/) is the drug regulatory agency in Uganda that is responsible for the effective regulation of drugs (including quality herbal medicines) and healthcare products to protect and promote Human and Animal Health (NDA, 2025). The NDA draws its power from Section 5(i) of the National Drug Policy and Authority Act, Cap. 206 of the Laws of Uganda (2000 Edition). The Ministry of Health is responsible for all policy review and development and for the supervision of health sector activities (MOH, 2025).

Registration of herbal drugs (referred to as Human Herbal Medicines) is regulated by the NDA. The Natural Chemotherapeutics Research Institute (NCRI), which is a constituent institute under the Uganda National Health Research Organisation (UNHRO), is a government research and development institute created by an Act of Parliament in 2015 and affiliated to the Ministry of Health (MoH) (NCRI, 2025). The NCRI plays a pivotal role in the registration process by conducting the laboratory analysis of all herbal products for product registration by the NDA to ensure that the products meet the optimum quality and safety standards appropriate for human consumption (NCRI, 2024).

To notify a locally made herbal drug in Uganda, herbalists must first ensure that the product meets safety and efficacy standards and then register it with the National Drug Authority (NDA). This involves assessing the plants used, demonstrating their safety through testing, and verifying the product’s effectiveness for its intended use.

### Zimbabwe

In Zimbabwe, there are two regulating authorities identified. The Medicines Control Authority of Zimbabwe (MCAZ) (https://www.mohcc.gov.zw/) is the main body responsible for the regulation of medicines, including herbal medicines. It is also responsible for its manufacturing, distribution, storage, registration, and sale. The MCAZ works in collaboration with the Ministry of Health and Child Care (MoHCC), which has overall oversight of health policy in Zimbabwe (MCAZ, 2015a; MOHCC, 2023). The Traditional Medical Practitioners Association (TMPA) is the regulating authority for the traditional medical practitioners under the Ministry of Health and Child Care. Its main responsibility is the registration and licensing of traditional health practitioners (MOHCC, 2023; Moyo, 2019). It is also involved in registering and controlling the sales of herbal medicines.

The MCAZ derived its mandate to evaluate all complementary medicines intended for public use in Zimbabwe following the passing of the legislation known as the “Medicines and Allied Substances Control (Complementary Medicines) Regulations, 2015” (MCAZ, 2015b).

#### Analysis of the comparison of the registration procedures of LMHMs in Nigeria, Ghana, Uganda, and Zimbabwe

Table 4 reports the comparison of registration procedures for LMHM in the four selected countries. In Ghana and Uganda, the initial stage for registering herbal products involves product analysis and validation. This is conducted in collaboration with accredited institutions. Analysis includes toxicology, microbiology, and phytochemical tests. In Nigeria, this stage involves business registration and facility inspection for good manufacturing practice (GMP) compliance.

In all four countries analyzed, the second stage involves an application submission procedure. Supporting documents to be submitted include proof of payment and an application form that requires detailed information about the herbal product, manufacturing process, and intended indication. In Nigeria, samples of the herbal product must be submitted for assessment.

In all four countries, review, screening, and evaluation of all submitted documents are conducted by the Drug Regulating Authorities (DRAs). In Nigeria, Uganda, and Zimbabwe, GMP inspection is conducted. In Nigeria, the laboratory analysis of the products is conducted by NAFDAC-designated laboratories in Lagos and Kaduna States. In Zimbabwe, a maximum of two review cycles will be considered during the evaluation process of applications before the registration verdict is finalized.

In Nigeria, upon approval, an herbal product is listed (simplified registration) and will be issued an NAFDAC registration number with a capital L at the end and must have a disclaimer “These claims have not been evaluated by NAFDAC.” In both Ghana and Uganda, upon approval, herbal products are notified (simplified registration). In Zimbabwe, herbal products are registered as either a complementary medicine for general sale or pharmacy complementary medicines. Herbal products are commercially available to the public.

After approval, listed or on notification, the DRAs in Ghana, Nigeria, and Uganda are subjected to pharmacovigilance or surveillance, where the herbal products are continuously monitored to ensure continued compliance with safety standards. In Nigeria and Uganda, the herbal product undergoes 2 years of pharmacovigilance for post-market surveillance. Products are recalled when complaints occur during pharmacovigilance. In Ghana, yearly and periodic renewals are being done for already registered products. In Uganda, after 2 years of product tracking, the herbal product is then fully registered with the NDA (Table 4).

A comparison of the main registration requirements for LMHM for the selected African countries is summarized in Table 5.

**TABLE 5:** Summary of the main registration requirements for locally manufactured herbal medicines in selected African countries.

Requirements	Ghana	Nigeria	Uganda	Zimbabwe
General minimum requirements (1)-safety	Scientific name and/or botanical names of the plants and parts of plants used. Quantity of active ingredients List of all excipients used and quantities per dosage units. Indications	Safety data on each plant component in the product: traditional use safety records and toxicity studies of each plant Formula and ingredients (plant and excipients/non-active ingredients and properties for formulation	Botanical identification/authentication. Ethno-medical information: documented proof of long period of use without evidence of safety problems (literature search/database). Toxicity studies. Dosage (posology). Dosage forms. Adverse reactions. Contraindications. Precautions	Botanical identification/authentication. Biological information (via literature search/database) on safety and efficacy of product: in the absence of published results of toxicological studies, documented experience of long-term use should form the basis of the risk assessment. Toxicity studies. Dosage (posology). Dosage forms. Adverse reactions. Contraindications. Warnings
General minimum requirements (2)-efficacy	Substantial evidence of the clinical effectiveness of the herbal medicinal product for the indications stated shall be required All indications and claims based on scientific evidence require human studies. Where only non-human data exist, indications and claims may be allowed on a case-by-case basis	Chemical constituents and record of use of the plant or polyherbal (if more than one plant): major uses, other uses, and claim verification (testimonials and anecdotal). Pharmacological properties: *in Vitro* and *in vivo* experiments (literature or experimental record)	Assessment of efficacy: the requirements for proof of efficacy will depend on the kind of indications for use and individual experiences as recorded in reports from physicians, traditional practitioners, or treated patients. Summary of claims. Active ingredients (where applicable)	Assessment of efficacy: the requirements for proof of efficacy depend on the kind of indications for use and individual experiences as recorded in reports from physicians, traditional practitioners, or treated patients. If use has not been documented, appropriate clinical evidence of efficacy is required. Active ingredients: the preparation of medicines whose active ingredients have been identified should be standardized. Evaluation of efficacy: for uses supported by clinical data, described in pharmacopoeias and other well-recognized documents, described in traditional medicine
General minimum requirements (3)-quality control	A certificate of analysis for each medicinal ingredient. Finished product specifications. Stability studies. Proposed shelf-life. Storage conditions. Physical/chemical identification tests. Microbial test according to the pharmacopoeia. Heavy metals. Pesticide residues. Test for foreign matter. Toxicological test	General description of each plant component contained in the product. Identification (phytochemistry) and standardization of herbal medicinal products *Formulation: formula and ingredients (plant and excipients/non-active ingredients and properties). Method of preparation/manufacturing process	Identification of Plant (s). Plant part used and condition of the plant material. General identity tests. Mandatory Purity tests Qualitative and quantitative composition of the active components. Quantity and type of excipients. Quantification of the active ingredient. Description of the process of manufacture. Specifications of the quality of the finished product. Methods of analysis. Stability studies. Licensing of manufacturing premises by the National Drug Authority. Packaging	Identification of plant(s). Part of the plant used, and the condition of the plant material used. General qualitative and quantitative tests of the plant materials. Purity tests. Specification for all excipients Name of the manufacturer. Description of manufacturing process and process controls (name and dosage form). Specifications of finished product. Analytical methods. Stability studies
Labeling information (package insert/leaflet)	No resemblance in spelling and pronunciation of name, or packaging to any previously registered product. Samples submitted to conform to existing labeling regulations	Dosage and frequency of administration. Route of administration. Contraindications. Side effects and adverse effects. Pregnancy and lactation (statement of exclusion). Overdose and antidote (if any). Drug–herb Interaction (record if any) Children below 12 years (statement of exclusion)—except formulation meant for children. Storage condition	Name of the product. Quantitative list of main active ingredients, including the common English name of the relevant plants. Dosage form. Therapeutic indications. Dosage. Overdose information. Contraindications, warnings, precautions, and major drug interactions. Manufacturing date. Expiry date. Lot/batch number. Name of the manufacturer or company with full address. Storage conditions. Main vehicle/base	The name of the FPP. Route of administration. A list of API. List of excipients. Instruction on use. The batch number. Manufacturing and expiry date. Storage conditions. Directions for use, and any warnings or precautions. The name and physical address of the manufacturing site(s). Name of the dosage form. The disclaimer “NO APPROVED THERAPEUTIC CLAIMS” on the label

#### Analysis of the comparison of the main registration requirements for herbal medicine products in Ghana, Nigeria, Uganda, and Zimbabwe

Table 5 reports the comparison of the main registration requirements for herbal medicines in the four selected countries. In all selected countries [Ghana, Nigeria, Uganda, and Zimbabwe], the regulatory authorities require botanical identification and toxicity studies. Also, a documented proof of long-term use without evidence of safety problems could serve as a requirement for safety and efficacy. Additionally, substantial evidence of the clinical effectiveness of the herbal medicinal product for the indications stated is required. Experiences recorded from physicians, traditional practitioners, or treated patients also serve as evidence for clinical efficacy.

General qualitative and quantitative tests of the plant materials and description of manufacturing process and process controls serve as evidence for quality control and GMP, respectively. In all countries, herbal products must conform to existing labeling regulations.

In Ghana, specific requirements stipulate that the herbal product must not resemble any previously registered product in the spelling or pronunciation of its name, or in its packaging. In Nigeria, certain requirements have been made mandatory for product registration. This includes information on the general description of each plant component contained in the product, major uses, other uses, and claim verification. Additionally, information on the formulation includes the formula, ingredients (plant and excipients/non-active ingredients), properties, and method of preparation/manufacturing process. All labeling information (package insert/leaflet) is also mandatory (Table 5).

#### Statements governing the conduct of clinical trials of locally manufactured herbal medicines as a prerequisite for their registration in selected African countries

In all the selected African countries [Ghana, Nigeria, Uganda, and Zimbabwe], the conduct of clinical trials is regulated by the DRAs [Ghana FDA, NAFDAC, NDA, and MCAZ, respectively], including clinical trials for herbal products. The general principles for conducting clinical trials of herbal medicine are similar to those of conventional medicines, although special considerations may apply in some cases.

LMHM, which is primarily based on traditional knowledge and practices passed down through generations within a community, is regarded as having a long history of use, serving as evidence for its safety and efficacy. Approval for product commercialization is granted without data from preclinical and clinical studies (Moreira et al., 2014).

Clinical trials are mandatory for new or relatively new herbal formulations, drugs for new indications, or new patient populations (NAFDAC, 2024). The DRAs provide guidance and approval for clinical trials, and no trial can commence without their written authorization.

The various guidelines for the conduct of clinical trials of herbal products that were accessed from the websites of various selected African countries ensure that all clinical trials that are to be conducted on humans, including those for herbal medicines, must be designed and conducted following sound scientific principles and ethical standards within the framework of good clinical practice. A clinical trial aims to ascertain the safety and efficacy of herbal products presented in modern dosage forms before they can be prescribed, dispensed, or commercialised for the public (NDA, 2020; FDA, 2024a; NAFDAC, 2021a; MCAZ, 2024).

The key guidelines and requirements include the following: approval by the DRA and Research Ethics Committee (REC), clinical trial registration on publicly available registries such as the Pan African Clinical Trial Registry (PACTR) or ClinicalTrials.gov., certifications for GCP and research ethics, and compliance with GMP. Botanical identification of plant parts and species, toxicity studies, and quality considerations to address potential contaminants such as herbicides, pesticides, and adulterants are unique features relevant to herbal products.

## Discussion

In this study, we compared the similarities and differences in the registration processes and minimum registration requirements of LMHMs of four African countries: Ghana, Nigeria, Uganda, and Zimbabwe. In all analyzed countries, the general emphasis is on those herbal products that are locally manufactured using indigenous medicinal plants and are submitted to the regulatory authorities for registration and subsequent market authorization. According to the guidelines for the registration of traditional medicines in the WHO African region, traditional medicines in categories 2 and 3 are most suitable for the study objectives. In brief, category 2 traditional medicines are widely used in the community. Its formulation is well known; it is prepared according to traditional methods, with safety and efficacy guaranteed by a long history of use. Category 3 traditional medicines are those developed through scientific research. The formulation is prepared in advance, containing active ingredients that are standardized extracts. The safety and efficacy are guaranteed by preclinical and clinical trials conducted using approved standard protocols. However, if medicines from categories 2 and 3 were to be made available on the market, they must meet the general minimum requirements for the registration of traditional medicines (WHO, 2010).

In herbal drug regulation, full registration entails a comprehensive evaluation of quality, safety, and efficacy, typically accompanied by strict requirements for documentation and evidence. The process requires a thorough application with detailed documentation, including manufacturing processes, quality control, and evidence of safety and efficacy. If the application is approved, the herbal medicine is granted registration and market authorization. Full registration often requires the conduct of clinical trials, approved by the regulatory body, following approved procedures. This is conducted to demonstrate the efficacy of the treatment claims of the proposed herbal product. These procedures are often prerequisites for full registration to obtain market authorization (NAFDAC, 2020a; Kaggwa et al., 2022).

In Ghana, Nigeria, and Uganda, the registration process provides a simplified pathway for LMHMs to be approved for marketing. In Nigeria, LHMHs are listed, whereas in both Ghana and Uganda, LMHMs are notified. In Zimbabwe, complementary medicines obtain approval as a process for registration (Usai et al., 2021; CCFU, 2008; NAFDAC, 2020a).

The procedures and definitions of the terminologies used for simplified registration (listing and notification) are country-specific. These are not global terms. Therefore, in Nigeria, NAFDAC’s “listing” for herbal products grants a “safe to use” status to products that meet the formulation composition, manufacturing, and labeling standards after a product review, but for which efficacy claims have not been scientifically evaluated. Listing is, therefore, a simplified, less stringent process of product registration than the full registration conducted for herbal products with no therapeutic claims. When a product meets all requirements, it is granted a listing status with a clear DISCLAIMER stating that “claims have not been evaluated by NAFDAC (2020a).”

In Uganda, notification for herbal products is a simplified regulatory process with the NDA for registration rather than a full registration. It is the process of informing the NDA of an herbal product before it can be sold, but it does not require a full evaluation of quality, safety, and efficacy. Due to the absence of herbal pharmacopeial methods to assess the quality of herbal medicinal products, the NDA often waives extensive toxicological evaluation and efficacy proof, thereby relying on the long history of use as evidence of safety. Market authorization of the product is granted without specific preclinical and clinical safety and efficacy studies once the evidence of a long history of use is established (Kaggwa et al., 2022). The notification process for the registration of herbal medicines ensures that products conform to safety and quality standards before they can be made available to the public (CCFU, 2008).

In the European Union (EU) Member states, a simplified registration procedure is offered for herbal medicinal products that require market authorization or product registration. This procedure states that once plausible efficacy data and sufficient safety data are demonstrated, clinical tests and trials on safety and effectiveness are not required (EMA, 2025). Additionally, Alostad et al. reported that for those herbal medicines in the UK, Germany, UAE, and the Kingdom of Bahrain that require a market authorization and evidence of proven clinical efficacy to be granted full registration as a medicine, a simplified registration is offered due to established traditional use, which serves as evidence of herbal product efficacy (Alostad et al., 2018).

The DRAs in all the analyzed countries conduct or collaborate in the review, screening, and evaluation of all submitted documents, including product laboratory analysis and GMP inspection. Collaboration with accredited research institutions like the Centre for Plant Medicine Research in Mampong (CPMR) in Ghana and the NCRI in Uganda provides credence and vigor to the assessment process. Potential risks posed by contaminants and adulterants can be minimized by implementing stringent testing procedures and quality assurance protocols. These ensure that herbal products are safe for consumption (Wang et al., 2023). Products must be free from contaminants and adulterants commonly found in herbal products, such as heavy metals, pesticides, microbes, fungi, and molds (Posadzki et al., 2012).

In Nigeria, Uganda, and Zimbabwe, GMP inspections of the manufacturing facilities are conducted to ensure compliance. GMPs are crucial guidelines in the herbal product industry, ensuring the quality of the product and protecting the health of consumers. They ensure the safety, quality, and consistency of herbal products (Wang et al., 2023).

In Nigeria, the inspection of herbal medicine facilities is conducted in accordance with the standard guidelines. GMP requirements for herbal medicines and nutraceuticals facilities include inspection in the areas of personnel, building/facilities, equipment, raw/packaging materials and sources, environmental sanitation, documentation, distribution systems and transportation, and handling. The procedure includes writing an application for inspection to be submitted to the Drug Evaluation and Research Directorate in the NAFDAC, along with supporting documents and scheduling of the facility for inspection. An unsatisfactory inspection outcome leads to the issuance of compliance directives and a pause in the process clock until the applicant responds satisfactorily to the directives (NAFDAC, 2021b). A study was conducted to assess the GMP compliance by manufacturers in Nigeria using a checklist of regulatory requirements to collect data from herbal medicine manufacturing facilities. The study reported an unfavorable response as none of the invited companies agreed to participate in the study. Various reasons could be responsible for their refusal to participate, including the reluctance of herbal medicine manufacturers to be monitored. This could be due to a suboptimal compliance with established regulatory requirements (Mponda et al., 2025). It has been emphasized in accordance with the provisions of the Food, Drugs, and Related Products Act Cap F33 LFN 2004 (formerly decree 19 of 1993) and the accompanying guidelines that no regulated product should be manufactured, imported, exported, advertised, sold, or distributed in Nigeria unless it has been registered (NAFDAC, 2021b). Therefore, despite the shortcomings, manufacturers adhere to the minimum requirements necessary for facility inspection for compliance with GMP so that they can register their herbal products.

Similarly, in Uganda, GMP inspection of all manufacturers of medicinal products by the NDA is a requirement for product registration. The National Drug Policy and Authority Act, Sections 2(d) and 5(e), mandates the NDA to exercise control on the manufacture, production, and quality of drugs (NDA, 2024). A study was conducted to evaluate the GMP compliance in herbal drug manufacturing in Uganda. The study utilized the reports obtained from the GMP inspections. A total of 36 reports were included. The results confirmed that an overall 1,236 deficiencies were made in the 36 inspections that were reviewed. In each facility, the number of deficiencies averaged 34.3 ± 15.8, with almost half of the parameters inspected being identified as nonconformances. The high level of nonconformance could be due to an inability to implement and maintain GMP requirements in a limited resource setting due to the high cost involved. The lack of knowledge of GMP requirements could also be responsible for the high level of nonconformance in the facilities reviewed. In conclusion, the study recommends a stricter facility inspection procedure during assessment. This is because the NDA, while conducting inspections, is urged to consider the government policy “Buy Uganda Build Uganda” to assist local indigenous businesses to compete favorably with imported goods, which results in less stringent assessments (Kwesiga et al., 2021).

In Zimbabwe, the MCAZ fully adopts and applies the current World Health Organization (WHO) GMP guidelines for all pharmaceutical and complementary medicine manufacturers. Before a product is registered and authorized for market in Zimbabwe, the manufacturing site must be deemed GMP compliant by the MCAZ inspectorate, which is mandated by the “Medicines and Allied Substances Control (Complementary Medicines) Regulations, 2015” to evaluate all complementary medicines intended for public use in Zimbabwe (MCAZ, 2015a).

Upon approval, a listed herbal product in Nigeria is issued an NAFDAC registration number with a capital letter “L” at the end and must have a disclaimer “These claims have not been evaluated by NAFDAC.” Various regions globally have their listed herbal products that are prevalent in that community. Hence, the capital letter L attached to the NAFDAC registration number assigned to a herbal product indicates that the herbal product is a listed drug in Nigeria and cannot be exported to other countries. Any herbal product meant for export must have a higher claim than what it is generally known to be used for in the community. In addition, the product must undergo a full registration process to ascertain claims and ensure product safety, efficacy, and quality.

In Uganda, all herbal medicinal products must be notified to the NDA before they can be commercialized through distribution, promotion, or advertisement. In addition, pharmacies and drug shops are not authorized to stock herbal medicines that are not notified with the NDA (NDA, 2017).

In Zimbabwe, herbal products are registered as either a complementary medicine for general sale or pharmacy complementary medicines. Complementary medicines, such as those registered as herbal products, are sold exclusively in pharmacies, whereas complementary medicines for general sale are not subjected to any restrictions on sale (Usai et al., 2021).

In Nigeria, both herbal products that consist of a single herb and polyherbal formulations undergo the same listing (simplified registration) procedure by the NAFDAC. An example of a listed herbal drug containing a single herb is the powdered leaves of *Guiera senegalensis*, commonly called sabara in the Hausa language. It has been used for centuries as an antimicrobial agent in the northern region of Nigeria. It has been listed and registered by the NAFDAC. Herb 25 is a product that consists of multiple herbs. Herb 25 is a NAFDAC-registered antimalarial herbal drug used to treat chloroquine-resistant malaria, and it is dispensed as tea bags (Muhammad, 2011). Additionally, Jobelyn®, a sorghum bicolor-based herbal supplement, is a herbal product that is used for its effect on oxidative stress markers in sickle cell disease in children. Jobelyn is available over the counter in community pharmacies in Nigeria (Ayuba et al., 2014).

The process of pharmacovigilance of LMHM requires post-market surveillance of the herbal drugs by the DRAs. In Nigeria and Uganda, approved herbal products are monitored for 2 years, whereas yearly and periodic renewals are implemented in Ghana. In Uganda, herbalists are responsible for post-notification product tracking to collect information or feedback on product safety and efficacy. In the absence of any negative feedback after 2 years, the information will be used to apply for a full product registration with the National Drug Authority (CCFU, 2008).

In Nigeria, despite the widespread use of herbal medicines, the status of pharmacovigilance is inadequate. There is inadequate safety monitoring from all stakeholders, including herbal medicine manufacturers, retailers, consumers, and regulatory authorities (Balogun et al., 2021). In Kampala city, Uganda, medically important bacteria were isolated from commercial herbal medicines, indicating the necessity for enhancing safety frameworks by the NDA (Walusansa et al., 2022). Furthermore, a study conducted by Frank et al. in Kumasi to determine the post-market safety and efficacy surveillance of herbal medicinal products from the consumers’ perspective recommended that regular large-scale post-market surveillance should be adopted throughout Ghana for appropriate documentation of safety, efficiency, and adverse effects on herbal medicinal products circulating in the market (Frank et al., 2019).

The minimum requirements for registering herbal medicines ensure product safety, efficacy, and quality. In all selected countries, emphasis is placed on safety and quality. Additionally, ethnomedical evidence, which is the experiences recorded from physicians, traditional practitioners, or treated patients, also serves as the proof for therapeutic efficacy. In all countries, herbal products must conform to existing labeling regulations. A study conducted in Mali reported on the contribution of using ethnomedical data to the marketing authorization of traditional herbal medicines. The study described the four categories of traditional herbal medicines as distinguished by their national regulations. Among the four categories, only traditional medicines in category 2 may be authorized for marketing based on ethnomedical evidence of a long history of use without the need for clinical trials (Sangho et al., 2024b).

A study conducted on the traditional medicine label reported that the Ghanian FDA mandates that all traditional herbal medicine products must meet the general labeling requirements that agree with the general guidelines provided by the World Health Organization (Agboka, 2021). These guidelines are similar to the requirements for labeling pharmaceutical medicinal drugs (FDA, 2024b). A study conducted to critically examine the labeling practices in herbal medicinal products reported that many labels were not in conformity with the national legislation in Belo Horizonte, Minas Gerais. Fifty-eight (58) products of the 150 samples analyzed had inadequate packaging, and only 21 samples provided vital information as contraindications. Information on the adverse effects of the herbal products was missing on the product labels of all the analyzed products (Faria and Labanca, 2024). These studies indicate a global lack of appropriate quality control measures, inadequate labeling, and the absence of suitable patient information, which further compromise the safety of herbal medicines (Ekor, 2014).

The principles governing the conduct of clinical trials of herbal medicine in all four countries are similar to those of conventional medicines; however, in some cases, special considerations may apply. A clinical trial is not required for the registration and subsequent market authorisation of some herbal products due to their long history of use (Moreira et al., 2014). Additionally, the tedious and costly process that is associated with the conduct of conventional clinical trials can be avoided by the good use of ethnomedical evidence for obtaining market authorization for traditional herbal medicines (Sangho et al., 2024b).

Our study reported simplified registration procedures by the selected African countries. The advantage of listing and notification of herbal medicines is to reduce the bureaucracy in registering herbal products by providing a simpler market authorization process (Usai et al., 2021). This applies to herbal drugs that have a known history of traditional use and are manufactured in accordance with GMP. Herbal drugs listed in the regulatory authorities’ registries must be quality-assured. They are notified and commercialized to allow early market access to local herbal products that have a low risk to the public (Usai et al., 2021). These less stringent registration procedures are beneficial, considering the requirements stated for a full herbal drug registration, as stated by the WHO for the African region. These requirements may be too difficult for the local traditional practitioner to meet. Generally, the registration requirements and procedures should be minimized while protecting the health of consumers (Ngcobo et al., 2012).

The role of regulators of herbal medicine in any country is to stimulate local industries to be able to compete favorably with imported complementary medicines. A study by Asase in Ghana reported the challenges encountered in the registration of herbal products due to difficulty in fulfilling the pharmacological data requirements for efficacy and safety as required by the Food and Drugs Authority—Ghana, which resulted in the registration failure of a significant number of herbal products (Asase, 2023). Likewise, traditional practitioners in Uganda expressed that the lengthy registration process, which is centralized and requires follow-up, is challenging and costly (CCFU, 2008). The MCAZ had only 2% of its registered drugs as local herbal products (Usai et al., 2021).

### Recommendation

Despite the positive attributes and the vital role of herbal medicine, the potential of herbal medicines has not been fully harnessed in African countries. Various factors must be addressed.

- Develop and strengthen regulations that address locally manufactured herbal medicines: these regulations should not be too difficult for the local traditional practitioner to meet. Generally, the registration requirements should be minimized while protecting the health of consumers (Ngcobo et al., 2012).
- Harmonization of herbal drug regulation within the African region: for herbal medicines to benefit from the African Continental Free Trade Area (AfCFTA), regulations must be harmonized so that it becomes easier to move products within the region. The herbal drugs that are listed in a certain community and can be exported to other regions can be commercialized within other African regions. Until there is harmonization, the regulations remain country-specific. Terminologies used in various countries could become continental terms with harmonization.
- Develop regulations on the requirement for the conduct of clinical trials that address the uniqueness of herbal medicines.

## Conclusion

The registration of locally manufactured herbal medicines is simplified in certain selected African countries. However, despite this flexibility, few LMHM products get registered and obtain market authorization. Drug regulatory authorities in Africa must address the various challenges that hinder the registration of herbal products and take further action to promote the local production of herbal medicines while complying with the fundamental registration criteria for product safety, efficiency, and quality control.

## Data Availability

The original contributions presented in the study are included in the article/Supplementary Material; further inquiries can be directed to the corresponding authors.
